# The Current State of Diagnosis and Treatment for Early Gastric Cancer

**DOI:** 10.1155/2013/241320

**Published:** 2013-02-28

**Authors:** Tomoyuki Yada, Chizu Yokoi, Naomi Uemura

**Affiliations:** ^1^Department of Gastroenterology, Kohnodai Hospital, National Center for Global Health and Medicine, 1-7-1 Kohnodai, Ichikawa, Chiba 272-8516, Japan; ^2^Department of Gastroenterology, National Center for Global Health and Medicine, 1-21-1 Toyama, Shinjuku-ku, Tokyo 162-8655, Japan

## Abstract

The prognosis for gastric cancer depends on its stage; so, detection in the early stage of disease is important, when complete and curative removal is possible. Accurate diagnosis can be facilitated by a sound understanding of the basic findings of white light endoscopy of early gastric cancer, and diagnosis can be refined further by the combined use of other imaging modalities such as image-enhanced endoscopy including chromoendoscopy and endoscopic ultrasonography. Minimally invasive endoscopic treatment has come to be the preferred therapeutic approach for early gastric cancer. In addition to conventional endoscopic mucosal resection, a new technique known as endoscopic submucosal dissection (ESD) has spread rapidly worldwide. Indeed, strategies for ESD have been established, devices developed, its indications expanded, and its safety and long-term results extensively reported. Some unique combination therapies involving endoscopy and surgical treatment have also been reported. It is anticipated that the number of patients undergoing endoscopic therapy will continue to increase, and the ongoing developments in endoscopic treatment are expected not only to improve gastric cancer prognosis but also to maintain good quality of life after treatment.

## 1. Introduction

Despite both the incidence and mortality rates of gastric cancer showing decreasing trends, gastric cancer remains one of the most common causes of death by cancer worldwide [1, 2]. There are significant regional differences in gastric cancer onset, with East Asian countries, including Japan and Korea, known to have a particularly high incidence rate compared to the Western countries. In Japan, following the introduction of a mass screening program that utilizes double-contrast barium radiography for early the detection of gastric cancer and alongside developments in endoscopic equipment and improved diagnostic capability, gastric cancer is now being detected more often in the asymptomatic stages [3]. As a result, approximately 50% of the cases of gastric cancer currently treated in Japan are early stage disease [4]. In contrast, in Western countries, gastric cancer is often detected at an advanced stage and prognosis remains poor. Prognosis depends on the stage at which it is detected, and complete excision of the cancer is the only curative option. The excellent postoperative results for early gastric cancer, with a 5-year survival rate of over 90% in both Western countries and Japan, indicate just how important it is to detect the cancer at the earliest possible stage [5, 6]. Moreover, with the improved detection rate of early gastric cancer in Japan, more minimally invasive treatments have been investigated, and the use of endoscopic mucosal resection (EMR) has become widespread. This technique has the support of many endoscopists, including those in Western countries [7]. In addition, a new modality of endoscopic treatment, endoscopic submucosal dissection (ESD), has become commonly performed in facilities across Japan, helping to dramatically increase the number of early gastric cancer cases treated endoscopically [8]. This paper will give a basic summary of early gastric cancer, outline the current state of its diagnosis and treatment, and summarize the prospects for future management of the disease.

## 2. Classification of Early Gastric Cancer 

**Figure 1:**
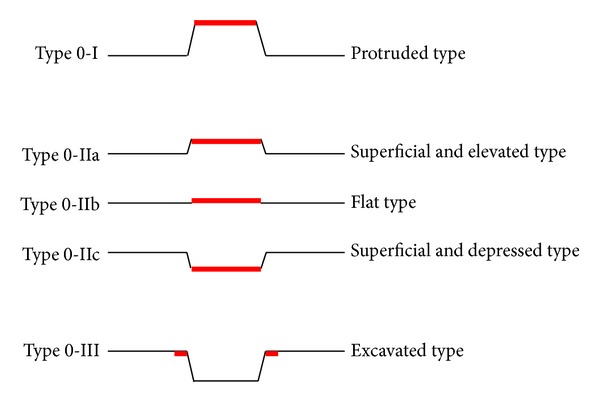
Macroscopic classification of early gastric cancer. (Type 0: superficial, flat tumor with or without minimal elevation or depression).

**Figure 2:**
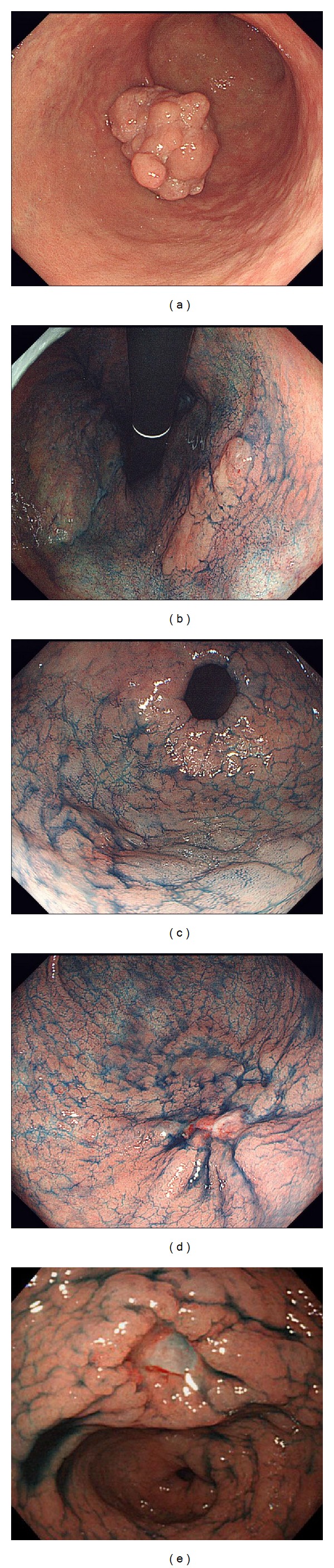
Macroscopic classification of early gastric cancer: (a) type 0-I (protruded type); (b) type 0-IIa (superficial and elevated type); (c) type 0-IIb (flat type); (d) type 0-IIc (superficial and depressed type); (e) type-III (excavated type).

Early gastric cancer is defined as remaining confined to the mucosa or submucosa, regardless of lymph node metastasis [9]. Japanese macroscopic classifications for gastric cancer are well known and divide early gastric cancer into three macroscopic types (0-I, 0-II, and 0-III) of which 0-II is then subclassified into 0-IIa, 0-IIb, and 0-IIc (Figures 1 and 2). The most common type of early gastric cancer is 0-IIc [10]. If two or more macroscopic types are mixed, the type that has spread further is recorded first. At an international workshop held in 2002, the Paris classification, which is based on the Japanese classification, was proposed and subsequently adopted as a simple and internationally unified classification for superficial gastrointestinal tumors [11].

Although cancer is pathologically diagnosed, the standards for pathological diagnosis are known to differ widely between Japan and Western countries [12]. In Western countries, cancer is diagnosed if the tumor has invaded the submucosa or muscularis mucosae and has at least invaded deeper than the lamina propria mucosae. In Japan, however, cancer is diagnosed based on cellular atypia or structural atypia, regardless of the extent of invasion. Although this discrepancy has lessened since the Vienna classification was proposed, lesions diagnosed as intramucosal carcinoma in Japan are still classified as high-grade adenoma/dysplasia (Vienna classification 4.1) in Western countries and often not diagnosed as cancer [13]. However, over 40% of lesions classified as equivalent to Vienna classification 3 or 4.1 on preoperative biopsy are diagnosed as cancer on the postendoscopic resection assessment, and it has been reported that a small number of cases are also accompanied by invasion into deeper regions of the submucosa [14, 15]. Therefore, when considering a complete cure, it is necessary to proceed carefully in cases with a histological diagnosis of neoplastic or dysplastic disease and, on case-by-case basis, to consider endoscopic resection.

## 3. Endoscopic Diagnosis

Unlike advanced cancer which is easy to detect endoscopically, early gastric cancer often appears as subtle changes in the mucosal surface. To avoid missing the presence of cancer on endoscopy, the characteristics of early stage disease must be well understood and gastric observation must be thorough and detailed [16]. While diagnosis is based on conventional white light endoscopy findings, the use of dye-based image-enhanced endoscopy (chromoendoscopy), equipment-based image-enhanced endoscopy (narrow band imaging—NBI), and endoscopic ultrasonography (EUS) is contributing to improved diagnostic capabilities for early gastric cancer.

### 3.1. White Light Endoscopy

Accurate diagnosis of early gastric cancer relies on having a good grasp of the characteristics of early stage disease and learning effective methods of endoscopic observation. When conducting white light endoscopy, it is important to pay attention to points such as slight color changes in the mucosa (pale redness or fading of color), loss of visibility of underlying submucosal vessels, thinning of and interruptions in mucosal folds, and spontaneous bleeding. Each of these findings is often a subtle change, and the endoscopist must be sure to clean off mucus adherent to the gastric wall, suction excessive gastric juice, supply sufficient air, and thoroughly observe the entire area within the stomach. Furthermore, when a lesion is observed, although a diagnosis of invasion depth is required, the endoscopist makes a comprehensive diagnostic judgment considering mural thickness and hardness, color, fold concentration, depression depth, and protrusion length. Reduction of air volume within the stomach is effective for observing wall thickness and hardness. The accuracy of conventional endoscopy to discriminate between intramucosal carcinoma and submucosal carcinoma is reported to be 72%–84% [17–19]. Furthermore, although high-definition endoscopy was developed to improve image quality and diagnostic capability, it also allows for more accurate diagnosis of early gastric cancer than ultrathin endoscopy [20].

### 3.2. Dye-Based Image-Enhanced Endoscopy: Chromoendoscopy

When subtle mucosal changes such as those described earlier are observed, detailed chromoendoscopic observation has been shown to effectively aid diagnosis. Typically, 0.1% indigo carmine is sprayed directly via the forceps channel. Early gastric cancer is diagnosed through comparison with the surrounding mucosa; so, the dye must also be sprayed widely over the mucosa surrounding the lesion. This method highlights subtle differences in elevation of the mucosal surface and changes in the surface structure and color and helps to improve qualitative diagnosis and diagnosis of the extent of invasion [21, 22]. Spraying of indigo carmine can, however, actually make the lesion boundaries unclear when mucus is adherent to the stomach wall; therefore, it is important to wash the lesion meticulously prior to spraying [23]. In addition, it should be noted that indigo carmine spraying can actually make it more difficult to observe the lesion if it exhibits few color changes or small differences in elevation.

### 3.3. Equipment-Based Image-Enhanced Endoscopy: Narrow Band Imaging

**Figure 3:**
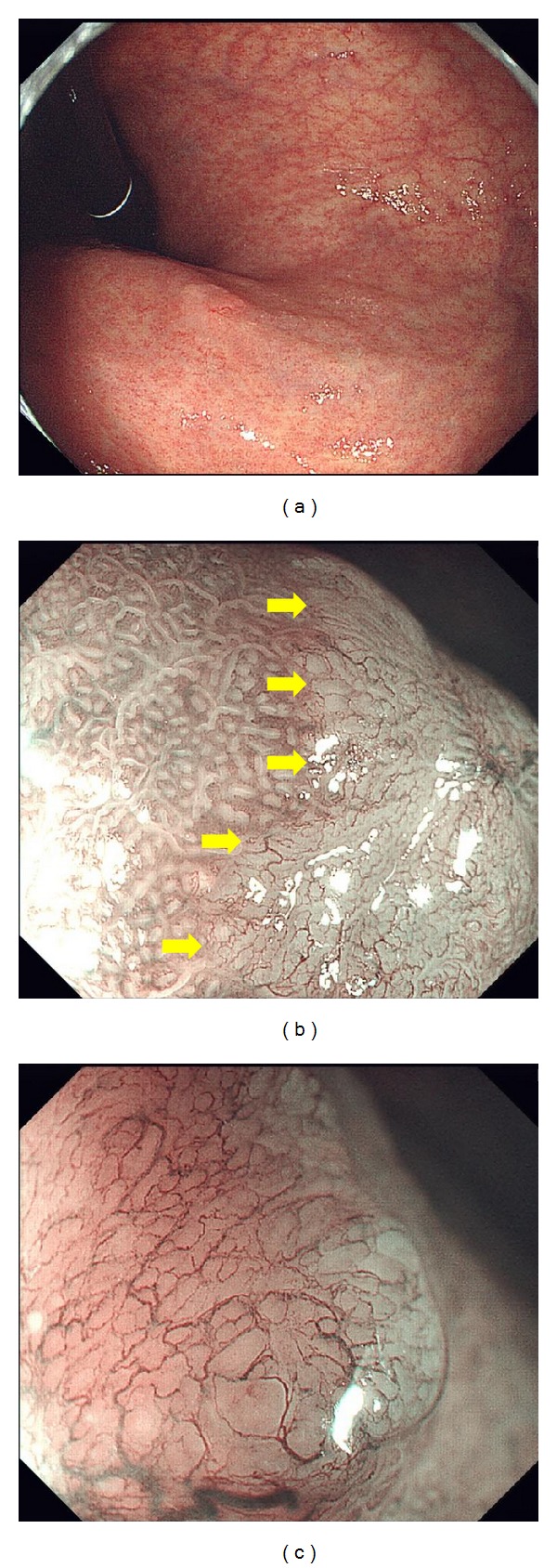
(a) A differentiated type 0-IIa lesion on the anterior wall of the middle gastric body. Magnifying endoscopy (ME) with narrow band imaging (NBI) shows (b) a clear demarcation line (arrows) at the border of the lesion with noncancerous mucosa to the left and cancerous mucosa to the right and (c) an irregular microvascular pattern plus the absence of a microsurface pattern in the cancerous mucosa.

NBI is a common type of equipment-based image-enhanced endoscopy that enhances the superficial surface structure and vascular architecture of the mucous layer by illuminating blue and green narrowband lights. Magnified endoscopy with NBI makes it possible to observe microvascular (MV) and microsurface (MS) patterns on the gastric mucosa in detail. Yao et al. combined changes in these patterns with the presence or absence of a demarcation line to establish a diagnostic system for gastric cancer called the VS classification system (Figure 3) [24]. This method has proven effective in actual clinical practice in two main areas.

The first area is differential diagnosis of small gastric cancer and gastritis. With conventional endoscopic observation, it has been difficult to discriminate between small gastric cancer and benign abnormalities (e.g., gastritis) accurately in many cases. A prospective randomized controlled trial that investigated differential diagnosis of small depressed gastric cancer and gastritis with depressed lesions of less than 1 cm proved that the accuracy with combined NBI magnification surpassed that with conventional white light endoscopy [25]. The trial revealed that when NBI magnification was conducted after conventional observation with white light, the diagnostic capabilities were excellent, with accuracy, sensitivity, and specificity all above 95%. The use of this method accurately distinguishes between cancer and gastritis, thereby reducing the number of unnecessary biopsies.

The second area is the utility of including NBI magnification for margin determination when conducting the detailed examination prior to endoscopic therapy. While securing lateral margins based on accurate diagnosis is crucial during endoscopic therapy, it is difficult to determine the margins for early gastric cancer with both conventional endoscopy and chromoendoscopy in approximately 20% of cases [26, 27]. Macroscopically flat types of lesions, such as type 0-IIb, are common in such cases; yet, combined NBI magnification can still help to improve margin determination capabilities [26, 28].

**Figure 4:**
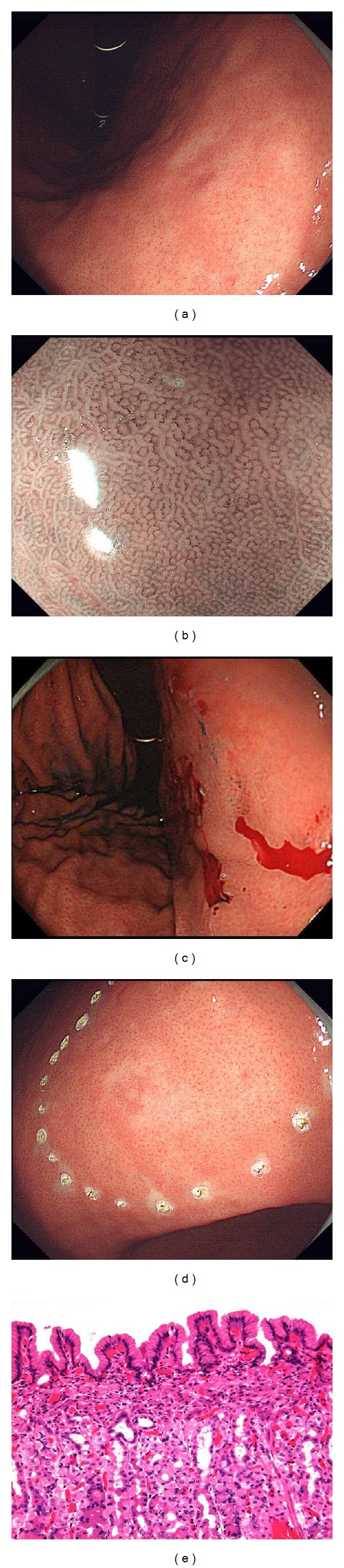
(a) An undifferentiated type 0-IIc lesion on the lesser curvature of the lower gastric body. (b) Magnifying endoscopy with narrow band imaging shows only a regular microvascular pattern plus a regular microsurface pattern in the cancerous mucosa with no identifiable demarcation line. (c) Multiple biopsies were taken from the mucosa surrounding the lesion to determine the horizontal extent of the cancer. (d) Marking dots were placed outside the scars of negative biopsies, and ESD was performed. (e) Histopathologically, the cancer extends to height of the glandular neck within the lamina propria mucosae and is covered with normal crypt epithelium.

Observation using NBI appears, therefore, to have clear advantages for the diagnosis of early gastric cancer if the necessary knowledge and skills are acquired. Endoscopists must be aware as NBI is not an all-purpose method. For example, because of the large size of the gastric lumen, use of nonmagnified NBI darkens the screen, limiting its use to the type of magnification outlined earlier. This means that it is not suitable for identifying lesions within the stomach. Furthermore, while it is very useful for margin determination with differentiated cancers, it has only limited utility with undifferentiated cancers. Undifferentiated cancers are often localized to the height of the glandular neck within the lamina propria mucosae, and the tumor surface remains covered with normal crypt epithelium. In these situations, concomitant use of NBI magnification will not reveal the characteristic irregular MV and MS patterns of cancer. Accordingly, diagnosis of the extent of undifferentiated cancers requires at least four negative biopsies in the area surrounding the cancer (Figure 4) [24, 26]. 

### 3.4. Endoscopic Ultrasonography

With more endoscopic procedures being performed for early gastric cancer, it has become necessary to determine preoperative invasion depth more accurately [29]. As mentioned earlier, although conventional endoscopic observation has an accuracy of around 80%, diagnosing invasion depth can be difficult in many cases. Furthermore, as such diagnosis with conventional endoscopic observation is subjective, EUS can be used to make a more objective diagnosis. Normally, the main objective of EUS for early gastric cancer is to determine whether the patient can undergo endoscopic therapy, and small diameter lesions are often targeted. Therefore, while the procedure is more often conducted using 20 MHz catheter-based miniprobes than with the 12 MHz catheter-based miniprobes, the latter are concomitantly used for large type 0-I lesions. The accuracy of depth diagnosis is reported to be 65%–86% and can reach 92% when normal endoscopic findings correspond to EUS findings [30–33]. However, these diagnostic capabilities drop for depressed lesions, undifferentiated cancers, cases accompanied by ulcers, cases of minute submucosal invasion, type 0-I lesions, and lesions located in the upper-third of the stomach. Accordingly, when it is difficult to diagnose lesion depth even with the combined use of EUS, endoscopic therapy can be conducted if there are no findings suggesting deep submucosal invasion, but doctors must judge whether or not to conduct curative resection on the basis of the pathological findings after endoscopic resection. Apart from the utility of EUS for diagnosing invasion depth, EUS can be used preoperatively to assess the submucosal vasculature in order to predict intraoperative bleeding during endoscopic therapy [34]. 

## 4. Endoscopic Therapy and Its Indications

In Japan, the 5-year postoperative survival rate for early gastric cancer excluding other causes of death is 99% for intramucosal carcinoma and 96% for submucosal carcinoma [35]. The frequency of lymph node metastasis in these patients is 3% for intramucosal carcinoma and 20% for submucosal carcinoma [36]. Given that favorable prognosis can be expected for early gastric cancer with the standard therapy of gastric resection accompanied by lymph node dissection, the indications for endoscopic therapy need to be strictly determined, as this therapy involves local excision and is not accompanied by lymph node dissection. Good indications for endoscopic therapy are, therefore, lesions where lymph node metastasis can be disregarded. In these cases, endoscopic therapy has significant advantages over surgery, as it is minimally invasive and preserves postoperative gastric function.

Since endoscopic mucosal resection using the strip biopsy method (two-channel method) was first introduced for endoscopic therapy in 1984 [37], various methods of EMR have been developed, including endoscopic resection with a cap-fitted panendoscope (EMRC). They are widely used in Western countries as well as Japan [7]. Lesions with an extremely low likelihood of lymph node metastasis have been identified by clinicopathological investigations of multiple surgery cases, and indications for endoscopic therapy have been compiled by the Japan Gastric Cancer Association [38, 39]. In principle, these indications state that the likelihood of lymph node metastasis is extremely unlikely and that the tumor should be of a size and in a site that allows it to undergo en bloc resection. Specifically, the lesion must be (1) a differentiated elevated intramucosal cancer less than 2 cm in size or (2) a differentiated depressed intramucosal cancer less than 1 cm in size without ulcer findings. However, due to the nature of the excision technique, which uses a snare, EMR also has the disadvantage that only a small area can be excised at a time, and so for larger lesions, it has higher rates of piecemeal excision and local recurrence [5, 40].

**Figure 5:**
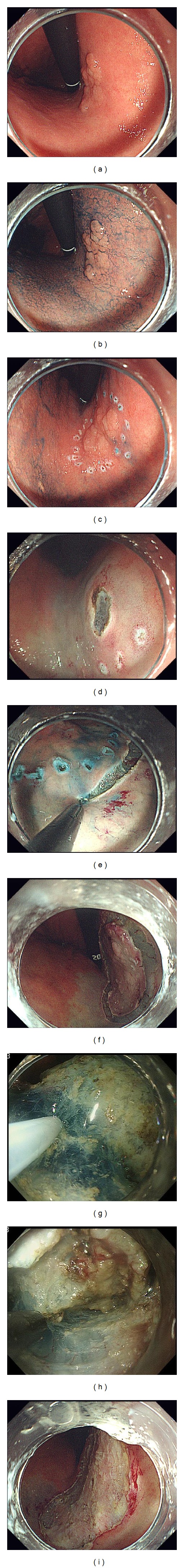
Procedure of endoscopic submucosal dissection. (a) Type 0-IIa lesion on the lesser curvature of the middle gastric body. (b) Chromoendoscopy with indigo carmine dye spraying. (c) Marking around the lesion. (d) Making small initial incision with a needle knife. (e) Mucosal cutting with an insulation-tipped diathermic knife-2 (IT knife-2). (f) Circumferential incision of the mucosa. (g) Additional submucosal injection of diluted epinephrine and indigo carmine. (h) Dissection of the submucosal layer with an IT knife-2. (i) The mucosal defect after resection of the lesion.

In this context, ESD was developed to make accurate en bloc resection possible. Indeed, ESD enables en bloc resection of large lesions, lesions with accompanying ulcer scarring, and lesions recurring after EMR because it involves dissecting along the submucosal layer directly using a high-frequency knife (Figure 5) [5, 41–44]. Following its introduction, investigations into additional indications for ESD have been made. In 2000, Gotoda et al. analyzed 5265 cases of early gastric cancer treated by surgery and reported that there was an extremely low risk of lymph node metastasis in cases that were (1) differentiated intramucosal cancers without ulcer findings, irrespective of tumor size, (2) differentiated intramucosal cancers less than 3 cm in size with ulcer findings, and (3) differentiated minute invasive submucosal (less than 500 *μ*m below the muscularis mucosa) cancers less than 3 cm in size [45]. Since then, the indications for ESD have been expanded to cover these lesions. With the accumulation of cases, there also appears to be an extremely low risk of lymph node metastasis for lesions that are undifferentiated intramucosal cancers less than 2 cm in size without ulcer findings, and these lesions are now indicated for ESD (Table 1) [46].

Although ESD is said to cause more complications than EMR, most instances of perforation or bleeding complications can be treated with endoscopy, and the risk for life-threatening complications is thought to be extremely low [47, 48]. Due to its widespread use for en bloc resection and low recurrence rate, a meta-analysis has shown ESD to be superior to EMR [49]. The continuing modification of endoscopic therapy devices and techniques and the spread of live demonstrations of ESD in Japan meant that many facilities are performing this technique nationwide and that the rate at which endoscopic therapy is being used for early gastric cancer is increasing dramatically [8]. There have also been reports, however, that conventional EMR and ESD produce the same results for lesions of less than 5 mm, and, rather than excising all lesions with ESD, EMR and ESD should be used properly weighing up the advantages and disadvantages [50]. Although ESD, like EMR, is now also conducted in Western countries, few cases of early gastric cancer are indicated for endoscopic therapy in the first place; so, it appears that it will take some time before it becomes widely used and for the techniques to become established [51, 52].

## 5. Postendoscopic Resection Management and Surveillance

Post-ESD management involves fasting on the day of the surgery in order to prevent delayed bleeding, resuming the intake of fluids from the following day and of food from the second day after surgery. Post-ESD ulcers require approximately 6–8 weeks to close completely, during which time antacids are administered [53]. Proton pump inhibitors (PPIs) are said to be more effective for preventing delayed bleeding than H2-receptor antagonists [54], and, recently, the combined use of mucosal protective antiulcer drugs with PPIs was reported to further promote ulcer healing [55, 56]. Long-term results for indicated lesions after EMR are favorable, with both the 5- and 10-year survival rates excluding death from other illnesses reported to be 99% [57]. It would appear then that if complete excision is achieved for an indicated lesion, the risk of local recurrence is extremely low, but it is desirable that patients undergo annual endoscopic surveillance to ensure early detection of metachronous cancer. Post-EMR/ESD metachronous cancer has a 3-year cumulative incidence rate of 5.9%, with a fairly high average time until initial detection of 3.1 ± 1.7 years [58]. 

With respect to the long-term ESD results for expanded indications, there is no difference in the 5-year survival rate of curative resection between the original and expanded indications [48, 59]. However, as there is currently insufficient evidence of the long-term results following curative resection under the expanded indications, these patients undergo annual endoscopic surveillance, in addition to half-yearly abdominal computed tomography or endoscopic ultrasonography, for at least 3 years in order to detect lymph node or distant metastasis.

When the pathological examination indicates noncurative resection, additional surgery including lymph node dissection is strongly recommended. The acceptability and safety of laparoscopy-assisted distal gastrectomy for early gastric cancer have been shown, and it is considered a first-line additional therapy after endoscopic resection in Japan [60–63]. Even in elderly patients aged 75 years or older, a noncurative resection group that underwent additional surgery had a higher survival rate than a noncurative resection group that underwent follow-up observation only. Thus, if patient activities of daily living and comorbidity allow, additional surgery can also be considered for elderly patients [64].

## 6. Prospects for the Future Management of the Disease

As the long-term ESD results under the expanded indications have yet to be established, the results of a prospective clinical trial by the Japan Clinical Oncology Group of its validity are eagerly awaited. If this study verifies the safety and effectiveness of ESD, the expanded indications may well become actual indications.

Although the number of early gastric cancer lesions that can be resected with endoscopic therapy is increasing, for nonindicated lesions, regular surgery remains the basic treatment. A large gap exists between these two treatment methods with regard to invasiveness, and minimally invasive surgical methods are being investigated to close this gap. One such method is ESD followed by laparoscopic lymph node dissection and histological evaluation [65]. Long-term results are now being reported, albeit only for a small number of cases, and this method may become an option for minimally invasive surgery in cases where lymph node metastasis cannot be confirmed or ruled out [66]. Furthermore, although the popularization of ESD has led to a clear decrease in lesions for which excision is technically difficult, lesions in the gastric fundus region or gastric corpus curvature or lesions with severe ulcer scarring can pose difficulties for ESD. These lesions have commonly been treated by radical surgery despite the extremely low likelihood of lymph node metastasis, but promising initial outcomes of laparoscopy-assisted full-thickness resection of the stomach, a minimally invasive surgical technique, have been published recently [67–70]. If sentinel node navigation is established for gastric cancer, it is likely that this minimally invasive surgical therapy will be developed even further.

## 7. Conclusion

Early detection and early treatment are vital, improving the prognosis of gastric cancer. As we have seen, endoscopic examination plays an important role in the early detection of gastric cancer, but in many cases, diagnostic accuracy is dependent on the skill of the endoscopist. First, it is important for endoscopists to conduct endoscopic observation carefully, fully aware that early gastric cancer is often indicated only by subtle changes on the mucosal surface. They must also acquire the necessary skills and knowledge to identify lesions on conventional white light endoscopy. In addition, the combined use of chromoendoscopy and image-enhanced endoscopy is expected to further improve diagnostic precision. Minimally invasive methods of treatment are currently the preferred treatment option for early gastric cancer, with the use of EMR, in particular, becoming widespread. At present, ESD is rapidly becoming favored in many countries including Japan as it shows an even greater potential for a complete cure. In the future, it is predicted that, with the development of technology and devices, endoscopic therapy will be performed more and more frequently, and that we will see more combinations of endoscopic therapy and surgery. A complete cure for early gastric cancer can be expected with radical surgery, but it is important to continue the development of minimally invasive endoscopic methods while also continually verifying their safety and complete curability. 

## Figures and Tables

**Table 1:** Criteria for treatment of early gastric carcinoma.

	Mucosal carcinoma				Submucosal carcinoma	
	No ulcer		Ulcer		SM1	SM2
	≦20	>20	≦30	>30	≦30	Anysize
Differentiated carcinoma	EMR/ESD	ESD	ESD	Surgery	ESD	Surgery
Undifferentiated carcinoma	ESD	Surgery	Surgery	Surgery	Surgery	Surgery
